# Corynoline alleviates renal ischemia-reperfusion injury by enhancing Nrf2/HO-1 pathway

**DOI:** 10.1590/acb406825

**Published:** 2025-08-18

**Authors:** Ming Chang, Buhe Sichen

**Affiliations:** 1 Inner Mongolia Medical University – Affiliated Hospital Hohhot – Department of Urology – Inner Mongolia Autonomous Region – China.

**Keywords:** Kidney, Reperfusion Injury, Oxidative Stress, Inflammation, Apoptosis

## Abstract

**Purpose:**

The potential of corynoline in ameliorating oxidative stress and inflammatory responses has been extensively demonstrated across various diseases. However, its specific role in the context of renal ischemia-reperfusion (IR) injury remains elusive. The aim of this study was to investigate the role of corynoline in renal IR injury and its mechanism.

**Methods:**

A rat model of renal IR injury was successfully developed. Samples of kidney tissue and blood were obtained to evaluate alterations in tissue damage, inflammatory response, oxidative stress, and apoptosis.

**Results:**

Corynoline significantly reduced renal IR injury, thus leading to improved renal function and mitigated tissue structure damage and cell apoptosis. Moreover, corynoline effectively suppressed the oxidative stress and inflammatory response induced by IR by increasing the superoxide dismutase (SOD) content, reducing the malondialdehyde (MDA) level, inhibiting neutrophil infiltration, and suppressing the release of proinflammatory cytokines. Mechanistically, corynoline successfully restored the expression of nuclear factor erythroid 2-related factor 2 (Nrf2) and heme oxygenase-1 (HO-1), which were significantly inhibited during renal IR injury. Furthermore, when coadministered with ML-385 (an Nrf2 inhibitor), the protective effect of corynoline against renal IR injury was counteracted.

**Conclusion:**

Corynoline protects against renal IR injury by suppressing oxidative stress, inflammatory responses, and apoptosis, and its mechanism of action may involve the activation of the Nrf2/HO-1 pathway.

## Introduction

Renal ischemia-reperfusion (IR) injury is an inevitable pathological occurrence in urological procedures, particularly concerning kidney transplantation^1^. IR results in impaired function of tubular epithelial cells, which subsequently contributes to the development of acute kidney injury, delayed graft function, and both acute and chronic rejection^1^,^2^. Hence, timely interventions during the progression of renal IR injury may enhance graft functionality and improve clinical outcomes for patients following kidney transplantation^3^. Unfortunately, there are currently no practical pharmaceuticals or techniques that are accessible for protecting the kidney against the consequences of IR injury. Despite ongoing efforts to fully understand the pathogenesis of renal IR injury, scientific investigations have revealed that oxidative stress, the inflammatory response, and apoptosis play pivotal roles in the molecular mechanisms of IR^3^,^4^. Hence, employing approaches that target inflammation and oxidative stress may be beneficial in managing renal IR injury.

Nuclear factor erythroid 2-related factor 2 (Nrf2) is a widely recognized transcription factor that plays a crucial role in maintaining redox balance, defending against cellular damage, and regulating the inflammatory response^5^,^6^. Nrf2 forms an inactive complex with its cytosolic inhibitor Kelch-like ECH-associated protein 1 (Keap1), which subsequently leads to the degradation of Nrf2 through proteasomes^6^. During inflammation or oxidative stress, Keap1 dissociates from Nrf2, which allows it to enter the nucleus and bind to an antioxidant response element (ARE). This binding prompts the transcription of genes that are responsible for producing antioxidants such as heme oxygenase-1 (HO-1) and superoxide dismutase (SOD). These antioxidants exhibit significant effects in combating oxidative stress, reducing inflammation, and preventing cell death, which serve as protective mechanisms for preserving kidney health under pathological conditions^7^,^8^. Numerous studies have highlighted the potential protective role of the Nrf2/HO-1 pathway in models of IR injury affecting various organs, such as the brain, heart, liver, and kidney. This pathway may contribute to the underlying mechanism of action for numerous therapeutic interventions and prodrugs that target ischemic conditions, including both natural compounds and synthesized chemicals^8^–^11^.

**Figure 1:**
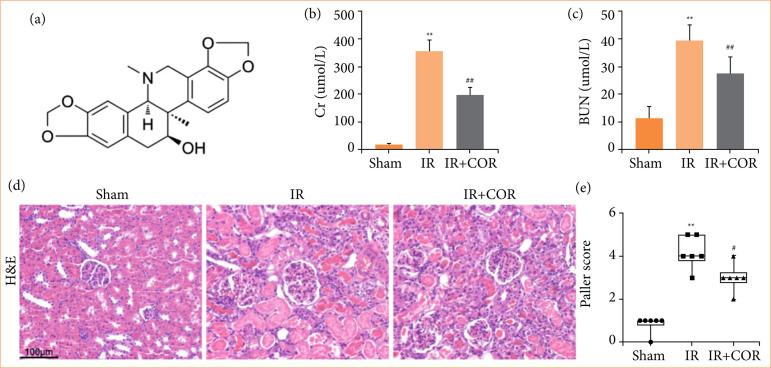
Corynoline alleviates renal IR injury in rats. **(a)** The chemical structure of corynoline. (**b** and **c**) Serum Cr and BUN levels. **(d)** Representative images of H&E-stained kidney tissue; scale bar = 100 μm. **(e)** Histological scoring of renal IR injury via the Paller’s score. All data are shown as means ± standard deviation (n = 6 per group).

IR: ischemia-reperfusion; COR: corynoline; Cr: creatinine; BUN: blood urea nitrogen; H&E: hematoxylin and eosin; **p < 0.01 versus the sham group; #*p* < 0.05 versus the IR group; ##*p* < 0.01 versus the IR group. Source: Elaborated by the authors.

Corynoline (Fig. 1a), which is derived from *Corydalis bungeana* Turcz., possesses various pharmacological properties, such as anti-inflammatory, antioxidative, and antitumor activities^12^–^14^. Recent research has demonstrated that corynoline can mitigate DSS-induced colonic injury by modulating the Nrf2/NF-kB pathway, thereby reducing the inflammatory response and oxidative stress^15^. However, the potential therapeutic effect of corynoline on renal IR injury remains unexplored. Hence, the aims of this study were to validate the protective role of corynoline in renal IR injury and investigate its mechanism involving the regulation of the Nrf2/HO-1 pathway.

## Methods

### Animals

Male Sprague-Dawley rats (250–300 g) were obtained from Beijing Vital River Laboratory Animal Technology Co., Ltd. (Beijing, China). These animals were kept in a controlled environment with a 12-hour light-dark cycle and had unrestricted access to food and water. The temperature and humidity of the housing facility were carefully regulated. All the animal care and experimental procedures adhered to the guidelines approved by the Animal Research Committee of Inner Mongolia Medical University (approval number: KY -2024019).

### Model establishment and experimental design

The rats were subjected to anesthesia via an intraperitoneal injection of sodium pentobarbital (40 mg/kg). Following the opening of the midline, microvascular clamps were applied to both renal pedicles of each rat in the four groups subjected to IR for 45 minutes. Reperfusion was subsequently performed for a period of 24 hours after the microvessel clamp was released. The rats were then randomly divided into five groups (with each group consisting of six rats) as follows:

- The sham group (sham): rats underwent only laparotomy without vascular occlusion;
- The IR injury group (IR): vehicle (5% DMSO, MCE, HY-Y0320) was intraperitoneally injected 1 hour before the operation, after which vascular occlusion was performed;
- The IR + COR group (IR + corynoline): corynoline (40 mg/kg, MCE, HY-N0705) was intraperitoneally injected 1 hour before laparotomy, after which vascular occlusion was performed;
- The IR + ML-385 group (IR + ML-385): ML-385 (an Nrf2 inhibitor, 30 mg/kg, MCE, HY-100523) was intraperitoneally injected 30 minutes before laparotomy, followed by vascular occlusion;
- The IR + COR + ML-385 group (IR + corynoline + ML-385): corynoline and ML-385 were intraperitoneally injected as described above, after which vascular occlusion was performed.

The solubilization of corynoline and ML385 was performed in a saline solution with 5% DMSO, followed by storage at 4°C. The selection of the injection dosage and timing for corynoline and ML-385 administration was based on previous investigations^12^–^14^. Following reperfusion for 24 hours, the rats were humanely sacrificed, and samples of blood and kidneys were collected for subsequent analysis.

### Serum creatinine and blood urea nitrogen levels

After the blood samples (5 mL) were subjected to centrifugation at 4,000 rpm for 10 minutes, a biochemical analyzer (Rayto, Shenzhen, China) was used to measure the levels of serum creatinine (CR) and blood urea nitrogen (BUN).

### Histological analysis and renal injury evaluation

Kidney samples were fragmented into small fragments and fixed in 4% buffered paraformaldehyde for 24 hours at room temperature. The samples were subsequently embedded in paraffin at 60°C for a period of 30 hours and subjected to staining with hematoxylin and eosin (H&E) for approximately 10 minutes. The severity of renal IR injury was evaluated based on the scoring criteria established by Paller et al., cited by Chen et al.^16^.

### TUNEL and immunohistochemistry staining

For the immunohistochemical analysis, kidney sections (5 µm) were subjected to overnight incubation at 4°C with primary antibodies, including an anti-MPO antibody (1:1,000, Abcam, ab208670); this was followed by incubation with a secondary antibody labelled with horseradish peroxidase. Subsequently, visualization of the antigen-antibody complex was achieved via 3,3’-diaminobenzidine tetrahydrochloride (DAB), and imaging was performed at a magnification of ×200 via Leica Microsystems. TUNEL staining was performed by using a one-step TUNEL apoptosis assay kit (Beyotime, China). Nuclei were stained with 2-(4-amidinophenyl)-6-indolecarbamidine dihydrochloride (DAPI) (Beyotime, China). The quantification and analysis of TUNEL-positive cells in kidney tissue were conducted in random fields of vision within the renal cortex at a magnification of ×200.

### Lipid peroxidation and antioxidant enzyme activity

A commercially available colorimetric assay kit (Nanjing Jiancheng Biology Research Institute, Nanjing, China) was used to assess the concentrations of SOD and malondialdehyde (MDA) in kidney tissues following the guidelines provided by the manufacturer.

### Western blotting

The protein fractions from the kidney tissues were obtained by homogenizing the tissue samples in RIPA buffer containing triple detergent (Solarbio, Wuhan, China). The proteins were then separated via 10% SDS-PAGE and transferred onto a polyvinylidene fluoride membrane (Bio‒Rad, Hercules, CA, United States of America) via electroblotting. Following a 2-h blocking step at room temperature with 5% skim milk, the membrane was incubated overnight at 4°C with a primary antibody; this was followed by incubation with the corresponding species-specific horseradish peroxidase-conjugated secondary antibody (1:5,000 dilution; Proteintech, SA00001-1/SA00001-2) for an additional 2 hours at room temperature. The primary antibodies that were used included anti-Nrf2 (1:1,000 dilution; ABclonal, A12444), anti-HO-1 (1:1,000 dilution; Proteintech, 10701-Ig), anti-Bax (1:1,000 dilution; ABclonal, A19684), anti-Bcl-2 (1:1,000 dilution; ABclonal, A0208), and β-actin (1:1,000 dilution; Proteintech, 666009-Ig) antibodies. Finally, the bands were visualized via enhanced chemiluminescence reagent (Wuhan Boster Biotechnology, Wuhan, China) and analyzed via ImageJ software version 1.51 (NIH, Bethesda, MD, United States of America).

### Quantitative real-time polymerase chain reaction

The Trizol reagent (Invitrogen, United States of America) was used for the extraction of total RNA following the manufacturer’s protocol. A Thermo Scientific RevertAid First Strand cDNA synthesis kit (GeneCopoeia, United States of America) was used to synthesize cDNA from the extracted total RNA. For reverse transcription-polymerase chain reaction (PCR) assays, the Applied Biosystems SYBR Green Mix Kit (Applied Biosystems, United States of America) was used. The primer sequences that were utilized in these experiments can be found in Table 1. To normalize the gene expression levels, the β-actin expression level served as a reference. Each sample underwent three analyses, and the data were processed via the 2^-ΔΔ CT^ method^11^.

**Table 1:** The primer sequences for quantitative real-time polymerase chain reaction.

Gene	Forward(5’-3’)	Reverse(5’-3’)
Nrf2	TGCCCTGGATATTCCCAGCC	CCTGCTGCTTGTTTTCCGTATT
HO-1	GGTGTCCAGGGAAGGCTTTA	TGGGGCATAGACTGGGTTCT
β-actin	AGATCAAGATCATTGCTCCTCCT	ACGCAGCTCAGTAACAGTCC

Nrf2: nuclear factor erythroid 2-related factor 2; HO-1: heme oxygenase-1. Source: Elaborated by the authors.

### Statistical analysis

The analysis was conducted via Statistical Package for the Social Sciences 15.0 statistical software (SPSS, Inc., Chicago, IL, United States of America). The normality of variance changes was determined using the Shapiro–Wilk normality test. Analysis of variance or nonparametric equivalent tests were performed, and post hoc tests were used for comparisons between groups. The obtained results were expressed as median with interquartile range or mean ± standard error of the mean, and a significance level of *p* < 0.05 was considered to indicate statistical significance.

## Results

### Corynoline alleviates renal ischemia-reperfusion injury in rats

To investigate the impact of corynoline on renal IR injury, we assessed the levels of serum CR and BUN in rats. These markers reflect changes in renal damage and metabolic function. Our findings demonstrated that rats subjected to IR exhibited significantly elevated levels of CR and BUN than those in the sham group (Figs. 1b and 1c). However, pretreatment with corynoline effectively reduced the levels of these serum markers in rats subjected to IR. Histological analysis via H&E staining revealed that, compared with the sham group, the IR group exhibited granular or vacuolar degeneration in renal tubular epithelial cells, along with partial or complete shedding of basement membrane epithelial cells. Notably, these pathological alterations were significantly mitigated following corynoline pretreatment (Fig. 1d). Furthermore, assessment via Paller’s renal pathology score revealed a significant increase in this score in rats in the IR group compared with those in the sham-operated group; however, this score was notably lower in the IR + COR group than in the IR group (Fig. 1e). These results indicated that corynoline reduces renal IR injury in rats.

### Corynoline reduces ischemia/reperfusion-induced renal oxidative stress and inflammatory response in rats

**Figure 2:**
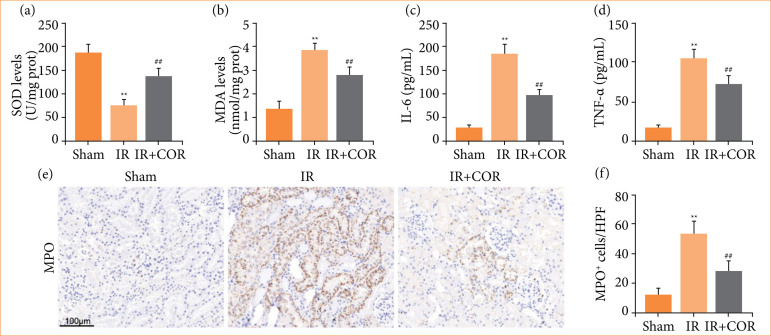
Corynoline reduces IR-induced renal oxidative stress and the inflammatory response in rats. (**a** and **b**) Concentrations of SOD and MDA in kidney tissue. (**c** and **d**) Levels of the proinflammatory factors IL-6 and TNF-α in serum. **(e)** Representative images of immunohistochemical staining for MPO; scale bar = 100 μm. **(f)** Quantiﬁcation analysis of MPO-positive neutrophils. All data are shown as means ± standard deviation (n = 6 per group).

IR: ischemia-reperfusion; COR: corynoline; SOD: superoxide dismutase; MDA: malondialdehyde; IL-6: interleukin-6; TNF-α: tumor necrosis factor-α; MPO: myeloperoxidase; **p < 0.01 versus the sham group; ##*p* < 0.01 versus the IR group. Source: Elaborated by the authors.

Oxidative stress and inflammatory response are two major detrimental events that contribute to tissue damage during renal IR injury^4^. We aimed to investigate the potential impact of corynoline on this process. As depicted in Figs. 2a and 2b, compared with the sham group, the IR injury group exhibited a significant reduction in SOD levels and a notable increase in the MDA concentration. Compared with the IR group, the IR + COR group demonstrated higher SOD levels and lower MDA concentrations. Furthermore, we observed significantly elevated expression levels of tumor necrosis factor-α (TNF-α) and interleukin- (IL-) 6 in the IR group compared with those in the sham group, whereas these proinflammatory cytokines were expressed at lower levels in the IR + COR group than in the IR group (Figs. 2c and 2d). Additionally, MPO staining was used to assess neutrophil infiltration within the renal tissue. As shown in Figs. 2e and 2f, compared with those subjected solely to IR, rats pretreated with corynoline exhibited markedly reduced neutrophil infiltration. These findings suggested that corynoline attenuates oxidative stress and inflammation in renal IR injury.

### Corynoline reduces ischemia/reperfusion-induced apoptosis in rats

**Figure 3:**
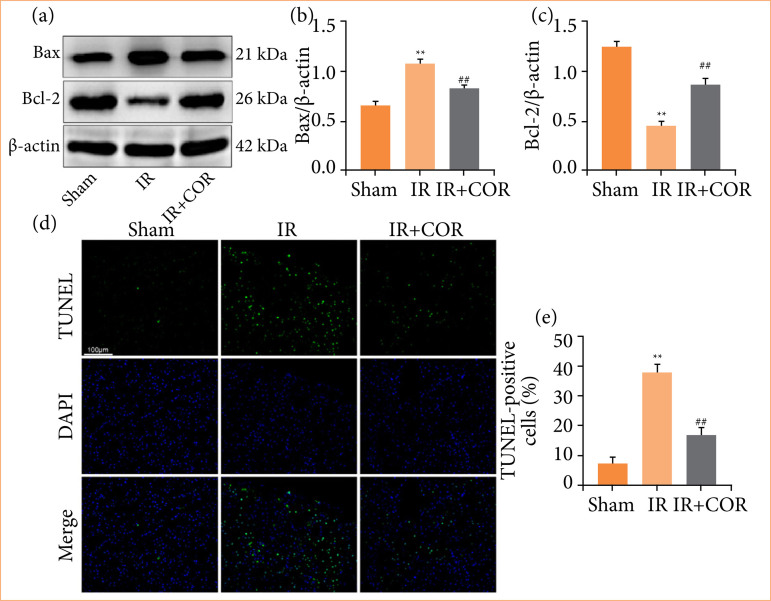
Corynoline reduces IR-induced apoptosis in rats. **(a)** Western blot analysis of Bax and Bcl-2 expression in kidney tissue. (**b** and **c**) Quantification of Bax and Bcl-2 expression normalized to that of β-actin. **(d)** Representative images of TUNEL-stained kidney sections. Scale bar = 100 μm. **(e)** Quantification of TUNEL-positive cells. The experiments were repeated at least three times. All data are presented as means ± standard deviation (n = 6 per group).

IR: ischemia-reperfusion; COR: corynoline; **p < 0.01 versus the sham group; ##*p* < 0.01 versus the IR group. Source: Elaborated by the authors.

We subsequently investigated the impact of corynoline on cell apoptosis triggered by IR in rats. As depicted in Figs. 3a–3c, Western blot analysis revealed that renal IR significantly increased the expression of the pro-apoptotic protein Bax and suppressed the expression of the anti-apoptotic protein Bcl-2 in this group of rats compared with those in the sham group. However, these effects were substantially reversed following corynoline pretreatment. Additionally, TUNEL staining revealed a greater number of TUNEL-positive cells in the IR group than in the sham group. Nevertheless, corynoline pretreatment reduced the number of TUNEL-positive cells induced by IR (Figs. 3d and 3e). These findings suggested that corynoline enhances anti-apoptotic activity and diminishes renal cell apoptosis during IR injury.

### Corynoline induces Nrf2/HO-1 activation during renal ischemia-reperfusion injury

**Figure 4:**
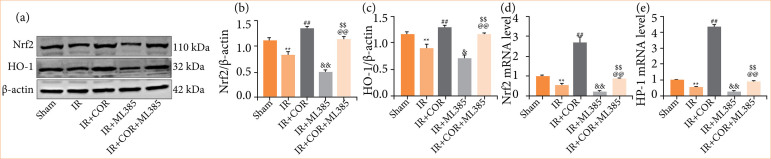
Corynoline induces Nrf2/HO-1 activation during renal IR injury. **(a)** Western blot analysis of Nrf2 and HO-1 expression in kidney tissue. (**b** and **c**) Quantification of Nrf2 and HO-1 expression normalized to that of β-actin. (**d** and **e**) Relative mRNA expression of Nrf2 and HO-1. The experiments were repeated at least three times. All data are presented as means ± standard deviation (n = 6 per group).

IR: ischemia-reperfusion; COR: corynoline; Nrf2: nuclear factor erythroid 2-related factor 2; HO-1: heme oxygenase-1; **p < 0.01 versus the sham group; ##*p* < 0.01 versus the IR group; ^&^ *p* < 0.05 versus the IR group; ^&&^ *p* < 0.01 versus the IR group; ^$$^ *p* < 0.01 versus the IR + COR group; ^@@^ *p* < 0.01 versus the IR + ML385 group. Source: Elaborated by the authors.

The Nrf2/HO-1 signaling pathway plays a crucial role in protecting against oxidative stress, inflammation, and cell death during renal IR injury^8^. Therefore, we investigated the expression of these signaling molecules during IR injury via Western blotting and quantitative real-time PCR (qRT-PCR) analyses. Our findings depicted in Figs. 4a–4e demonstrate that the levels of Nrf2 and HO-1 were reduced in kidney tissue following renal IR injury. However, corynoline pretreatment significantly increased the expression of Nrf2 and HO-1. Furthermore, ML-385, which is a specific inhibitor of Nrf2, notably decreased the levels of both Nrf2 and HO-1 expression. Interestingly, this reduction was partially reversed by corynoline pretreatment. These results suggested that the activation of the Nrf2/HO-1 pathway may be essential for facilitating the beneficial effects of corynoline.

### Blockage of the Nrf2/HO-1 pathway abolishes the protective effect of corynoline on renal ischemia-reperfusion injury

**Figure 5:**
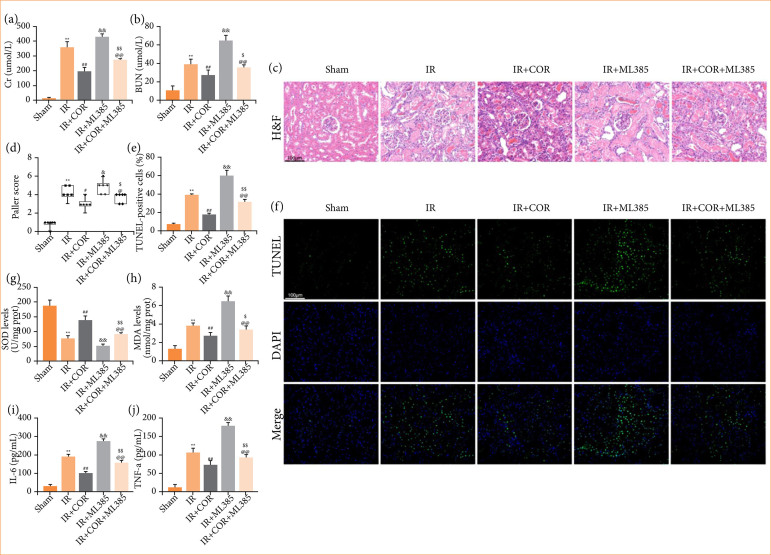
ML385 abolishes the protective effect of corynoline on renal IR injury. (**a** and **b**) Serum Cr and BUN levels. (**c** and **d**) Representative images of H&E-stained kidney tissues and quantitative analysis of renal injury represented as the Paller’s score. (**e** and **f**) Representative images of TUNEL-stained kidney sections and quantitative analysis of TUNEL-positive cells. (**g** and **h**) Concentrations of SOD and MDA in kidney tissue. (**i** and **j**) Levels of the proinflammatory factors IL-6 and TNF-α in the serum. All data are presented as means ± standard deviation (n = 6 per group).

Based on the findings, our hypothesis was that corynoline may play a protective role in renal IR injury by activating the Nrf2/HO-1 pathway. To investigate this hypothesis, we used ML-385 to obstruct the Nrf2/HO-1 signaling cascade. As anticipated, kidney injury was significantly exacerbated in the IR + COR + ML-385 group compared with the IR + COR group, as evidenced by notable increases in Cr and BUN levels, TUNEL-positive cells and injury scores (Figs. 5a–5f). Furthermore, ML-385 counteracted the corynoline-induced increase in SOD levels and reduction in MDA levels. Additionally, ML-385 inhibited the inhibitory effects of corynoline on IL-6 and TNF-α release (Figs. 5g–5j). These results indicated that ML-385 pretreatment diminished the protective effect of corynoline.

IR: ischemia-reperfusion; COR: corynoline; Cr: creatinine; BUN: blood urea nitrogen; SOD: superoxide dismutase; MDA: malondialdehyde; IL-6: interleukin-6; TNF-α: tumor necrosis factor-α; H&E: hematoxylin and eosin; **p < 0.01 versus the sham group; #*p* < 0.05 versus the IR group; ^##^ *p* < 0.01 versus the IR group; ^&^ *p* < 0.05 versus the IR group; ^&&^ *p* < 0.01 versus the IR group; ^$^ *p* < 0.05 versus the IR + COR group; ^$$^ *p* < 0.01 versus the IR + COR group; ^@^ *p* < 0.05 versus the IR + ML385 group; ^@@^ *p* < 0.01 versus the IR + ML385 group. Source: Elaborated by the authors.

## Discussion

Renal IR is known to cause tissue damage during kidney transplantation and negatively impacts the functional recovery of the graft, thus often resulting in an unfavorable prognosis^2^. The underlying mechanism of renal IR injury is highly intricate, with accumulating evidence suggesting that excessive oxidative stress and an inflammatory response significantly contribute to its pathogenesis^4^. Hence, it may be beneficial to explore strategies aimed at reducing both the inflammatory response and oxidative damage as potential approaches for mitigating renal IR injury. The results of the present study provide evidence that corynoline has a renoprotective effect against renal IR injury by mitigating oxidative stress and suppressing the inflammatory response and apoptosis. The underlying mechanism of action may involve the activation of the Nrf2/HO-1 pathway. Consequently, corynoline demonstrates promise as being a novel therapeutic intervention in clinical settings.

The sterile inflammatory response is a crucial component of renal IR injury^17^. Neutrophils play a pivotal role in this process, as their recruitment induced by IR can trigger the release of proinflammatory cytokines such as TNF-α and IL-6, thus leading to renal tissue damage^18^,^19^. Therefore, the inhibition of the infiltration of inflammatory cells and the production of inflammatory factors could mitigate kidney injury caused by IR. Previous research has demonstrated that corynoline can suppress the production of inflammatory cytokines in LPS-stimulated RAW264.7 cells^12^. Moreover, another study suggested that corynoline effectively inhibited neutrophil influx, MPO activity, and the release of the inflammatory cytokines IL-1β, TNF-α, and IL-6 in mice with LPS-induced acute lung injury^13^. Similarly, our findings indicated that corynoline pretreatment significantly reduced TNF-α and IL-6 release during renal IR injury while also mitigating neutrophil recruitment, thus indicating that corynoline can attenuate inflammatory responses during renal IR injury.

In the context of renal IR injury, an imbalance between antioxidant defense systems and reactive oxygen species (ROS) generation leads to oxidative stress^20^. SOD and MDA are integral components of cellular antioxidant defense mechanisms against oxidative stress^21^. MDA serves as a marker for lipid peroxidation and indirectly reflects tissue oxidative stress levels20. SOD is an essential enzyme that plays a crucial role in combating free radical attacks^22^. Previous studies have demonstrated that corynoline has the potential to reduce ROS production and lipid peroxidation in the colon tissues of DSS-treated mice and alleviate oxidative stress injury in rats with rheumatoid arthritis induced by Freund’s adjuvant arthritis (CFA) by increasing glutathione (GSH), catalase (CAT), and SOD activities while reducing MDA levels^15^,^23^. Consistent with these findings, our experimental results indicated that corynoline pretreatment increased SOD activity and decreased the MDA concentration during renal IR injury. Therefore, corynoline was able to attenuate oxidative stress-mediated injury during renal IR injury.

Apoptosis, which is a type of cellular death, has been demonstrated to occur following renal IR and is characterized by the activation of either extrinsic or intrinsic pathways^24^. Upon stimulation by IR, a significant amount of ROS is generated, which disrupts the balance between oxidation and antioxidation systems^25^. Endogenous ROS-induced tissue damage can lead to cellular dysfunction and trigger a cascade of inflammatory responses, thus ultimately resulting in cell necrosis and apoptosis^24^. Bax and Bcl-2 play crucial roles in regulating cell apoptosis. Bax promotes apoptosis by relocating to mitochondria and forming pores in the inner membrane. In contrast, Bcl-2 acts as an antiapoptotic factor that is essential for cell survival by inhibiting the release of cytochrome C from mitochondria into the cytoplasm, thereby preventing caspase activation and subsequent apoptosis^26^,^27^. In this study, we observed that IR had opposite effects on the expression levels of Bax and Bcl-2; specifically, it upregulated Bax expression while downregulating Bcl-2 expression, thus indicating that IR can activate apoptosis. Importantly, corynoline pretreatment effectively counteracted the effects of IR on these two proteins associated with apoptosis regulation, thus suggesting that corynoline has defensive capabilities against renal IR injury through the inhibition of apoptosis. The protective effect of corynoline against renal IR injury was further confirmed via TUNEL staining, which revealed fewer TUNEL-positive cells in the group treated with both IR and corynoline than in the group treated with only IR. These findings are consistent with changes observed in peripheral circulation parameters reflecting renal function, such as decreased serum CR levels.

To explore the potential mechanisms underlying the protective effects of corynoline against renal IR injury, we examined the expression levels of Nrf2 and HO-1 in kidney tissues. The Nrf2/HO-1 pathway is known to enhance the cellular defense system against oxidative damage and inflammation (which has been linked to various diseases and oxidative stress responses), such as by reducing renal IR injury^28^–^30^. In addition, further in-depth research from relevant studies has revealed that this pathway not only regulates oxidative stress but also plays a crucial role in regulating apoptosis and inflammation^28^,^31^. Many natural compounds have been found to alleviate inflammatory injury and oxidative stress in renal IR injury by modulating the Nrf2/HO-1 pathway^32^,^33^. Importantly, a previous study showed that corynoline inhibited the LPS-induced inflammatory response in RAW264.7 cells by activating the Nrf2 signaling pathway^12^. Our study revealed that rats with renal IR injury exhibited decreased expression levels of Nrf2 and HO-1, which were rescued by corynoline pretreatment. To further investigate the correlation between corynoline and the Nrf2/HO-1 pathway, we used ML-385, which is a widely utilized inhibitor of Nrf2, to suppress the activity of the Nrf2/HO-1 pathway. Our findings revealed that cotreatment with ML-385 diminished the protective effect of corynoline against renal IR injury. Additionally, ML-385 exacerbated renal IR injury; however, corynoline counteracted the detrimental impact of ML-385. These findings suggest that corynoline mitigates renal IR injury by activating the Nrf2/HO-1 pathway.

## Conclusion

In summary, this is the first study to demonstrate the therapeutic potential of corynoline in treating renal IR injury in rats. Corynoline has a protective effect on the kidneys by modulating the Nrf2/HO-1 pathway, thus leading to reduced inflammation, oxidative stress, and apoptosis. Although further investigations are warranted, these findings suggest that corynoline demonstrates promise as being a viable treatment option for renal IR injury.

## Data Availability

The data will be available upon request.
